# Human Centred Design Considerations for Connected Health Devices for the Older Adult

**DOI:** 10.3390/jpm4020245

**Published:** 2014-06-04

**Authors:** Richard P. Harte, Liam G. Glynn, Barry J. Broderick, Alejandro Rodriguez-Molinero, Paul M. A. Baker, Bernadette McGuiness, Leonard O’Sullivan, Marta Diaz, Leo R. Quinlan, Gearóid ÓLaighin

**Affiliations:** 1 School of Engineering and Informatics, Department Electrical & Electronic Engineering, NUI Galway, University Road, Galway, Ireland; E-Mails: rich1874@gmail.com (R.P.H.); barry.broderick@nuigalway.ie (B.J.B.); alejandro.rodriguez@nuigalway.ie (A.R.-M.); gearoid.olaighin@nuigalway.ie (G.OL.); 2 Galway Connected Health, NUI Galway, University Road, Galway, Ireland; E-Mails: liam.glynn@nuigalway.ie (L.G.G.); bernadette.mcguinness@nuigalway.ie (B.M.); 3 General Practice, School of Medicine, NUI Galway, University Road, Galway, Ireland; 4 Technical Research Centre for Dependency Care and Autonomous Living, Neàpolis Rambla de l'Exposició, 59-69 08800 Vilanova i la Geltrú, Spain; E-Mail: marta.diaz@upc.edu; 5 Centre for 21st Century Universities, (C21U) Georgia Institute of Technology, 760 Spring Street Atlanta, GA 30331-0210, USA; E-Mail: pbaker@cc.gatech.edu; 6 Department of Medicine, School of Medicine, NUI, Galway, University Road, Galway, Ireland; 7 Enterprise Research Centre, University of Limerick, Castletroy, Limerick, Ireland; E-Mail: Leonard.OSullivan@ul.ie; 8 Physiology, School of Medicine, NUI, Galway, University Road, Galway, Ireland; E-Mail: leo.quinlan@nuigalway.ie

**Keywords:** eHealth, ageing adult, elderly, medical devices, human-centred design, human computer interaction, usability, human factors, user experience, user acceptance

## Abstract

Connected health devices are generally designed for unsupervised use, by non-healthcare professionals, facilitating independent control of the individuals own healthcare. Older adults are major users of such devices and are a population significantly increasing in size. This group presents challenges due to the wide spectrum of capabilities and attitudes towards technology. The fit between capabilities of the user and demands of the device can be optimised in a process called Human Centred Design. Here we review examples of some connected health devices chosen by random selection, assess older adult known capabilities and attitudes and finally make analytical recommendations for design approaches and design specifications.

## 1. Introduction

When designing healthcare products (systems, devices and services), knowledge of the end users’ capabilities and expectations are key design considerations. In order for a product to be successful, these considerations must be addressed before and during the design process. For a new product where no brand loyalties exist, accurate knowledge of how end users will interact with the product may be the key factor separating it from rival offerings. This knowledge can also eliminate design problems and reduce potential user frustration before product release [1].

Usability, User Experience and Human Factors are all concepts that refer to how a user interacts with a product and how it should be conceived and developed to provide a satisfactory experience to the end-user. Usability is a property which describes the extent to which a product can be utilised by users to achieve specific goals effectively, efficiently and satisfactorily in a particular context. A usable product is easy to use, easy to learn how to use and easy to remember how to use. The concept of usability was first employed in 1983 for software design and has since been adopted for many kinds of interactive product designs [2]. Human Factors (HF) is the field describing human capabilities and constraints, investigating human features, structures and processes involved in interacting with designed artefacts and environments. HF provides models and knowledge to feed the process of developing products that fit human requirements. The basic sciences on which HF is based are physiology, anatomy, cognition and affective and social psychology. User Experience (UX) is the experience provided by using a product or service. UX encompasses not only the functionality related aptness, addressed by product usability, but the affective and hedonic dimension of ownership and use. A positive User Experience provides the user with feelings of pride, value or self-efficacy while on the other hand a negative User Experience can generate feelings of frustration, disability or stigmatisation. The most widely used definitions of the above terms are summarised in Table 1.

The three terms described in Table 1 are similar but each term can be clearly distinguished when put into context. However, the relationship between all three is not so easily distinguishable. Usability and human factors should be considered the main components of user experience. Table 2 presents some example observations of the aspects of usability and human factors associated with the use of everyday products and how these affect user experience.

**Table 1:** Definitions of terminology employed in user centered design.

Term	Source of Definition	Definition
User Experience	ISO 9241-210 [3]	1 …*a persons’ perceptions and responses that result from the use or anticipated use of a product, system or service* 2 …*all aspects of the user’s experience when interacting with the product, service, environment or facility*
Usability	ISO 9241-11 [4]	*“…the extent to which a user can use a product to achieve specific goals with effectiveness, efficiency and satisfaction*.…
Human Factors (Ergonomics)	ANSI/AAMI HE75 2009 [5]	*“…the application of knowledge about human capabilities (physical, sensory, emotional, and intellectual) and limitations to the design and development of tools, devices, systems, environments, and organizations”*

**Table 2:** Common devices and the inter-related roles that usability and human factors play in creating a positive or negative user experience.

System/Device/Service	User Experience (UX): What is the overall impression and response?	Usability: How easy is it to use?	Human Factors: How does it look, feel, sound?
Water Faucet	Positive	User is able to turn on the tap and control temperature, on-time and power without hesitation and without instruction	Finish on the taps affords comfortable and effective grip; no great force or awkward physical movement is required to operate the tap
	Negative	Unintuitive controls; no means to effectively control power and on-time; no natural mapping of functions	Sharp edges on taps, slippery surface; user must exert unnecessary force to activate controls
Car Rental Website	Positive	User can freely navigate menus and can navigate intuitively to where they want to go, errors are limited and are easily reversed	Buttons, links and lists are clearly visible, font size is easy to read, colour scheme is agreeable, excessive clicking is minimalised
	Negative	Options are not clearly presented; users have to randomly explore to find correct paths. User has to depend on search bar/help menu	Font is difficult to read, colour schemes make it difficult to process information, users need many clicks to complete tasks
Blood Pressure Measurement Device	Positive	User can put on device easily and quickly initialise measurement through button press or switch; intuitive feedback from display	Font on screen is easy to read; screen brightness is adequate; button requires little force to operate; alarms or beeps are clearly audible and adjustable;
	Negative	Device is not easy to put on; Measurement sequence does not initialise easily or quickly; readings takes too long to show on screen; no audio/tactile feedback	Buttons and strappings are cumbersome and uncomfortable, alarms beeps are too faint or too loud, screen text is difficult to read;

In Section 1 we set the scene of Human Centred Design, its general role in healthcare and connected health design. In Section 2 we explore the importance of Human Centred Design considerations with connected health devices specifically with reference to some commonly used devices used by older adults. In Section 3 we detail the older adult user capabilities and the changes in perception, cognition, psychosocial and psychomotor performance that occur with ageing. In Section 4 we look at the challenges and design approach that is recommended when designing for older adults. Finally in Section 5 we concluded on the benefits of Human Centred Design guidelines in providing a comprehensive framework for the role of usability, human factors and user experience in the design of any product.

When a product is assessed on how it performs in terms of usability, human factors and user experience, a very comprehensive and thorough analysis of how acceptable the product is to users can be made. This paper will identify the key requirements to meet these issues for connected health devices specifically for the older adult population.

### 1.1. Human Centred Design: An Umbrella Term

Human Centred Design (HCD) is a multi-stage design process which is heavily focused on human factors engineering, usability engineering and user experience optimisation. Therefore, HCD can be used an umbrella term to describe how the three terms defined in Table 1 are incorporated into the design process. Furthermore HCD also recognises the importance of incorporating as much user input and user testing into the process as early and as often as possible. The definition of HCD is outlined in the ISO standard *Human Centred Design for Interactive Systems: ISO 9241-210* (Table 1) [3]. The term “Human” is used as opposed to “User” in order to acknowledge product stake holders that may not be users and as such the term HCD will be now be used throughout this paper [6]. The guidelines of Human Centred Design as per the guidelines in ISO 9241-210 are as follows:

(a) Understand and specify the context of use
(b) Specify the user requirements
(c) Produce design solutions
(d) Evaluate

### 1.2. The Importance of HCD in Healthcare

Humans are prone to errors and some level or instance of error is sometimes unavoidable during technology interaction. Technology must be designed, especially in safety critical situations, to reduce the chance of making an error while also providing the opportunity to recognise and recover from errors when they are made. The use of technology in the field of medicine and healthcare can compromise safety if the product does not meet high HCD standards. For example, in a usability study of a hand held device for filling out prescriptions it was found that usability associated errors with the device directly contributed to the wrong medication being prescribed to patients [7]. Usability errors included incorrect data entry and screen object selection errors. A study of mortality rates before and after the implementation of a Computerised Physician Order Entry (CPOE) showed that mortality rates had in fact increased since the implementation of the system, with data entry related errors cited as a major factor [8]. These examples and others [9,10], have served to heighten the awareness of HCD and how its successful incorporation into healthcare technologies is of paramount importance.

A lack of adherence to HCD during development can lead to a product recall. For example in a very recent case, a prescription infusion pump (Hospira Symbiq, Hospira Inc., Lake Forest, IL, USA) used to deliver a range of therapeutic agents either by intravenous, intra-arterial or epidural means was recalled by the FDA due to an error with the touchscreen interface [11]. The problems would be familiar to anybody who has experience with a low-medium quality smartphone or a touchscreen kiosk. Sometimes the touchscreen would not respond to user selection, would produce a delayed response or would register a different value from the value selected by the user. Failure of the touchscreen to respond appropriately to user input resulted in delays and interruptions in therapy as well as excess delivery or under delivery of medication.

The advantages of optimizing device design through application of HCD extend beyond improved safety. An FDA report on the importance of Human Factors and usability engineering in medical device design concluded that many device manufacturers have found that the application of a user centred approach in the design of their products reduces the need for modifications and costly updates after market introduction and offers competitive advantages [12]. The report also added that “*With increased safety, the likelihood of your incurring expenses associated with product recalls or liability is reduced; when Human Factors Engineering/Usability Engineering approaches are used in the design of devices, particularly if the perspective of users is taken into account, the overall ease of use and appeal of a device can simultaneously be enhanced*.”

### 1.3. Connected Health

With healthcare technology in the home, HCD becomes even more critical as patients could be using devices without supervision. Connected health is a term used to encompass healthcare concepts such as eHealth, telehealth, telemedicine, smart home technology (SHT), digital health and remote care. These terms all refer to the use of health technology to deliver effective healthcare to patients remotely. The first connected health centre was founded in Massachusetts General Hospital by Joe Kvedar who defined it as the use of messaging and monitoring technologies to bring care to where the patient is, when the patient needs it [13]. An increasing focus on reducing healthcare costs for patients of all ages has spurred the growth of the connected healthcare market. Connected health is allowing people to independently take control of their own healthcare, all the while enjoying the comfort of their own home. In a study by Geisenger Health Plan it was found that using connected health monitoring post-discharge for heart patients reduced readmission to hospital by 44% [14].

**Figure 1:**
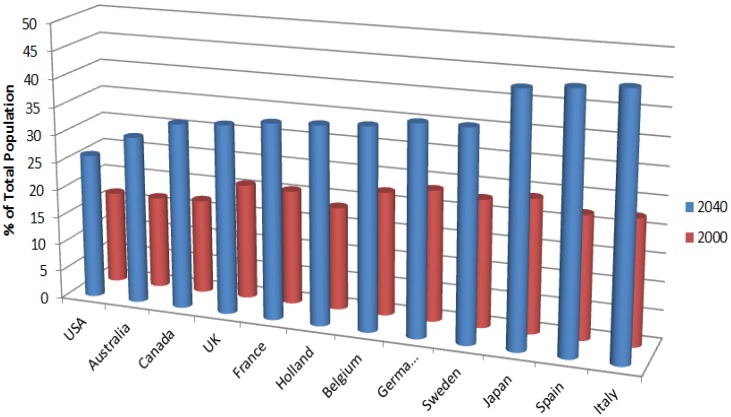
Percentage of people over the age of 60 years as percentage of total population by country.

A primary user group of connected health products are older adults and the need for smart technologies which can provide safe and independent healthcare for this increasing demographic has been one of the main driving forces behind the connected health revolution. It was estimated in 2002 that the world p older adult population, those aged 65 or more, will increase by more than three times by 2050 [15]. In the future there will be more older adults, both in absolute numbers and as a percentage of the population. The older population is growing faster than the total population in practically all regions of the world (Figure 1) [16,17]. This population group is also more likely to live with multiple chronic diseases [18].

The need for connected health products which can provide effective healthcare for the older adult is considerable given these demographic projections. The success of the connected health model and the impact that it can have on people’s lives depends on the design of smart usable products that meet high standards of human factors, usability and user experience. These standards can be met most effectively through the pursuit of Human Centred Design.

## 2. Connected Health Devices for the Older Adult

There is a vast range of connected health devices currently available today which are used by the older adult. These devices share many common features; they are typically compact, electronic modules that carry out at least one specific healthcare function. They generally have buttons, switches, screens and speakers *etc.* and are designed to measure some aspect of a person’s health status. There may be different levels of interaction, both in terms of complexity and regularity, across a range of devices. It would be useful to identify how the user currently interacts with typical connected health devices. We have randomly selected a range of commercially available today, commonly used connected health devices and examined some of their features in the context of the capabilities of the older user.

### Common Personal Connected Health Devices

Many connected health devices share common features (Table 3). Glucometers for blood glucose measurement, usually consists of a device module and an accompanying lancing tools. The lancing tool is loaded with a one use only sterile lancet and cocked, usually by pushing or twisting the base of the pen and also has a feature for setting the depth to which the lance will pierce the skin. Blood pressure monitors typically consists of an inflatable cuff which is wrapped around the arm or wrist with or without a hand held module which displays both systolic and diastolic blood pressure and heart rate. A pulse oximeter is intended for the non-invasive measurement of arterial blood oxygen saturation and pulse rate. Typically it uses two LEDs (light-emitting diodes) generating red and infrared light. The display typically shows both the percentage of oxygen in red blood cells (SpO2) as well as the pulse rate. Lung function can be measured using a peak flow meter or spirometer, which measure air flow and lung volumes respectively. Peak flow is measured by simply blowing sharply into the tube and reading off the embedded scale. Some models have indicator lights that illustrate good or bad results. More modern spirometery devices such as the Spirodoc have multiple built in tests available for comprehensive remote respiratory analysis. The device is the latest in smart home health technology, complete with a touch screen interface. The device has similar functionality and interface to a common smartphone as well as similar weight and dimensions. This kind of device also has a built in activity monitor which can correlate level of activity with respiratory assessment providing information on peak flow and lung volume. Portable ECG scanners are used within the connected health framework to check pulse and to monitor ECG output. The HCG-801 E from Omron is a common example of a portable ECG recorder. Although any weighing scale can be used to record weight at home, the latest in connected health weight devices allow readings to be sent to any device via Bluetooth. The PMP4 scale from Omron is such an example. Body temperature reading is an important part of health monitoring. There are various forms of thermometer available as connected health devices. Ear thermometers such as the GentleTemp from Omron are capable of producing an instant read and are convenient for all types of user. Under arm/oral thermometers such as the I-Temp from Omron work simply by placing the tip of the device in the appropriate site and waiting for 60 s, before taking the reading from the LCD display. A pedometer is a continuous monitoring device for measuring step count. It is a useful way to establish activity levels in a given day and over more prolonged periods. Although now commonly available on smartphones, standalone pedometers such as the HJ-720ITC from Omron are still widely used for both casual sports and health care management.

**Figure 2:**
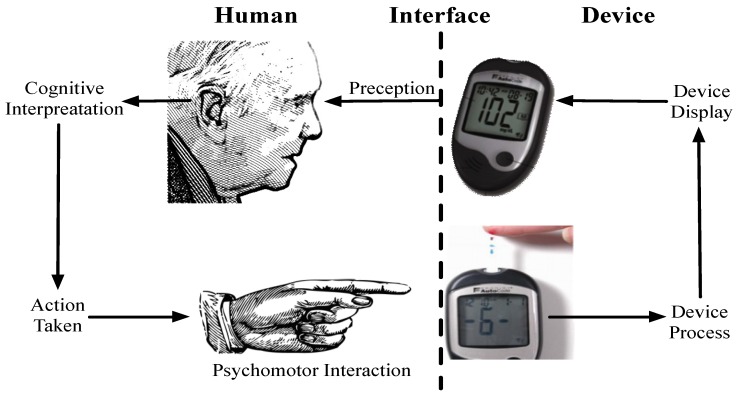
The general framework of human machine interaction can be applied to connected health devices such as a blood glucose metre.

In relation to the kind of connected health devices listed in Table 3, the general framework of human machine interaction still applies where the user perceives information from a display/device (limited by perception abilities), they process the information to form an impression of the device state (limited by cognitive abilities), they then physically interact with the device (limited by psychomotor skills) this process is illustrated in Figure 2.

Thus, effective interaction by the user with the connected health device requires that the demanded perceptual, cognitive and psychomotor elements associated with the device do not exceed the skills of the user. As the normal aging process impacts on perceptual, cognitive and psychomotor skills, it is clear that the skill level demanded by the device must be carefully designed to reflect this change. This is the basis of Human Centred Design. With proper application of HCD, the design of a device can be modified to be either less dependent on the abilities of the user or more accommodating of changing capabilities. The next section will characterise the older adult user group by discussing and highlighting the various changes that occur in terms of perceptual, cognitive and psychomotor abilities as one ages.

**Table 3:** Examples of Connected Health Devices, the typical input and outputs of the devices and the general level of interaction required.

Connected Health Devices	Functional Analysis	Device Controls	Device Output Elements
Blood Pressure Monitor	User must sit still in an upright position and place the cuff on the bare skin ensuring that the arm is in such a position that the cuff height is level with the heart. User reaches and presses the start button on the unit when complete readings will then appear on screen. The displayed value is read, interpreted, and acted upon.	Buttons, arm/wrist cuff	Screen symbols and alpha/numerical characters, audible tone indicators, light indicators
Glucose Metre/Lancet	Device is powered on via main button. The lancing device is cocked and the depth set. The head of the device is pressed against the skin and a button is pressed which fires the lancet. Blood sample is placed on a test strip and inserted in the device. Blood glucose level in the sample is measured and value is displayed on screen, audio feature also reads out measurements. The displayed value is read, interpreted, and acted upon.	Buttons, insertion of plastic strip, depth gauge on lancet	Audio tones and verbal feedback, alpha numeric screen characters
Blood Oxygen Monitoring	Power on the device is typically initiated by simply placing device over the finger tip. Once aligned reading will commence and take a matter of seconds. The fingernail must be right under the LED lights and the finger must be kept still during the measurement. Readings will be displayed on screen. The displayed value is read, interpreted, and acted upon.	Button for power, Finger input	LED light, small screen with alpha numeric characters
Pedometer	Device is initiated using main power button. Variables such as the weight and stride length of the user must be inputted. The device is placed in a pocket, a closely held bag or attached to the belt. Readings are displayed on screen. Audio feedback can also indicate when certain milestones have been reached. The displayed values are read, interpreted, and acted upon. Most devices can store a number of days of measurements and are USB enabled to upload data to a computer.	Buttons for input settings and power	Screen, beeps, small screen with alpha numeric characters, screen symbols, some models with verbal feedback
Spirometer	Unit is powered on via power button and users input their anthropometric details. User can carry device around like a pedometer To enter spirometry mode the user simply clips on the mouthpiece and selects the required spirometery test from the user menu. User breathes into the mouthpiece as per the instructions on the display. Test results are displayed on screen. The displayed values are read, interpreted, and acted upon. User can save the reading on the device under their name or upload it to a computer for further software manipulation via USB or Bluetooth.	Breathing input mouthpiece, buttons, Spirodoc is touchscreen	Screen display, graphical readings, alpha numeric characters, audible tones to signify breathing test sequences
Weighing Scales	The device pairs up automatically with any available Bluetooth device. To initiate the reading the user has only to step onto the scales. The scale calibrates and produces a reading within 3 s and automatically sends the reading to a nearby device via Bluetooth. The displayed values are read, interpreted, and acted upon.	Stand on scales, calibration/mode change possibly required with buttons	Reading appears on screen numeric display, voice feedback
Thermometer	Device initiated by pressing the On Measure button. Place probe under tongue or in arm pit. When the reading is ready, the device will emit a tone to indicate reading complete, the displayed values are read, interpreted, and acted upon.	Buttons, placement of metallic strip at indicated site	Tones to signify reading, numerical output on screen
ECG Scanner	Powered on by pressing power button on the front of the device. User presses their index finger on the metallic electrodes on one end of the device and then presses the other end of the device against their chest. User presses the start button and must hold position for 30 seconds for the measurement to complete. Readings are displayed on screen. The user is asked whether they want to store the data. The display will show the ECG waveform, the heart rate and a letter from a-m corresponding to what the waveform reading entails about the condition of the heart. The displayed values are read, interpreted, and acted upon.	Power and settings button, placement of finger on metallic strip	Tones to signify reading, alpha numerical characters on display

## 3. The Older Adult User

The rapid evolution of connected health has been primarily in response to the increasing need to deliver effective healthcare to the homes of an expanding population of older adults. Before proceeding it is important to define the terms ageing and older adult. Aging refers to the biological, psychological, and sociological changes occurring in human beings as they advance in chronological age [19]. Age related changes in the ability to detect, interpret and respond to visual and auditory information are often sufficient to compromise performance on a wide range of daily tasks [20,21]. These deficits are sometimes profound but more often are moderate in degree. There is some ambiguity as to what defines an older adult in terms of age given the different rates of change exhibited by individuals. As such chronological age is useful only as an indicator of changing social roles [22]. In the developed world, chronological age plays a prominent role in classifying older adults as a population group. The age of 65, roughly equivalent to retirement age in most developed countries is said to be the beginning of old age although many developing countries it is seen to begin at the point when active contribution is no longer possible which may be a more fitting definition [23]. The rate of age-related change is also a function of other factors such as environment, training and the effects of chronic disease and indeed multi-morbidity which is the rule rather than the exception in this population [24].

Numerous studies have explored the potential role of technology to help motivate older adults to adopt a healthier lifestyle. The use of mobile devices and real time computing to collect and provide appropriate information can assist users in managing their own healthcare and to motivate them to improve their lifestyles [25]. This is particularly true in the management of conditions such as obesity, diabetes and heart disease [26]. In terms of activity management, pedometers have been shown to help establish reasonable and visible goals for increasing the physical activity levels of older adults [27]. The same has been shown for wearable accelerometers [28]. Smart home technology can provide two-way communication that can be used for monitoring, health alerts, and other services. Designing technology for the older adult user requires greater effort in understanding the distinctive needs and capabilities of the end user. It is suggested that designers should become familiar with the effects of ageing at several levels [29]. Older adults are a diverse population group with extremely varying degrees of ability and for the most part, are an independent age group in terms of daily living and the associated tasks. There are a range of other factors that influence if a technology will be adopted, often in spite of it demonstrable benefits to users health [30,31] While beyond the scope of this review consideration should also be given to why some user don’t choose the healthy option when it is available and why more people don’t use existing proven technologies. A challenge for designers and current older users is their technology generation which is based more commonly in mechanical and electro-mechanical equipment. In the not too distant future we can expect an internet generation of older adults which will no doubt have implications for gerontechnological adoption [32].

**Figure 3:**
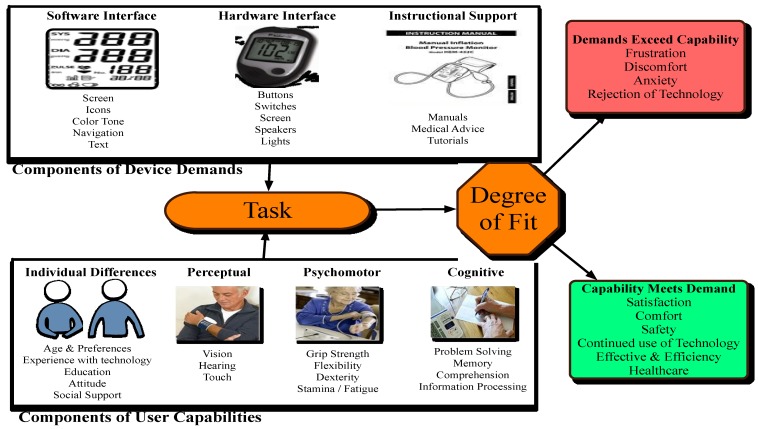
User capabilities *versus* device demands.

In Section 2 (Common personal Connected Health Devices, Table 3) examples and scenarios of use for common connected health devices which the older adult population may utilise are presented. User capabilities will vary across chronological age in terms of their perceptual, cognitive and psychomotor capabilities and users will respond differently to the demands of the device depending on these capabilities. A useful framework of capability *versus* demand is shown in Figure 3 [33].

The framework identifies the user components of the device that will create a demand on the perceptual, cognitive and psychomotor capabilities of the user. One of the goals of the design process from a HCD point of view is to create a balance between demand and capability in order for a product to reach a high degree of acceptance. This balance is also referred to as degree of fit. The user capabilities outlined in Figure 3 may well change with the chronological age of the user and as such the design of connected health devices for the older adult population must be carefully considered. We have already addressed the scenarios of use for various connected health devices. We will now identify what kind of changes a person might expect to their user capabilities given a change in perceptual, cognitive and psychomotor abilities.

### 3.1. Perceptual Changes with Ageing

Perception refers to the function of the physical senses such as sight, hearing and touch, smell and taste. In the context of device interaction sight, hearing and touch are the three senses that are responsible for the majority of the interaction with the surrounding environment.

#### 3.1.1. Vision

Nearly all interactions with connected health devices involve dynamic visual activities. A measurable degree of vision loss is inevitable as a person ages. Visual acuity is the term used to describe the clarity or sharpness of vision, and can be assessed under different environmental (lighting) conditions. There are many components to functional vision that are utilised during human machine interaction (Table 4). A comprehensive study of 900 subjects between the age of 58 and 102 carried out by Brabyn *et al.*, illustrates the different rates of decline for each of the visual components listed [21]. Table 4 shows the “normal young” values for each of the components and then shows the factor by which the components will have generally deteriorated for each of the age groups. As reported in Table 4 high contrast acuity, the standard measure of vision, declines very little even into very old age. The median value for the oldest group is no more than a factor of 2 worse than visual acuity for young adults with a steep a noticeable depreciation only occurring after the age of 75. Components such as LCALL and LCAG show a sharp deterioration after the age of 75.

**Table 4:** Measures of Vision performance under different conditions and effect of aging. Numbers indicate the factor by which visual components decline from normal.

Component	Description	Age Profile							
		Normal	60–65	65–70	70–75	75–80	80–85	85–90	90–95
Low Contrast Acuity (LCA)	The clarity of vision when viewing low contrast surfaces, for example grey scale images are considered low contrast.	20/27	1.1	1.2	1.5	1.8	2	3	4
High Contrast Acuity (HCA)	The standard measure of visual performance is taken by measuring high contrast acuity.	20/20	1	1	1.1	1.2	1.3	1.5	2
Low Contrast Acuity in Low Luminance (LCALL)	Similar to LCA except in poorly lit environment. Home lighting can be as much as 4 times dimmer than a work or office environment.	20/40	1.5	1.7	2	2.5	3	4	6
Acuity in Glare (AG)	The ability to focus vision when competing light sources are present in the environment, this is sometimes referred to as disability glare.	20/40	1.9	2	2.5	3	3.5	6	18
Colour Discrimination (CD)	A person’s ability to distinguish between objects or lights having different colours.	10 (D-15 Score)	1	1	1	1	1	2.5	5
Contrast Sensitivity (CS)	A person’s ability to visually distinguish an object that is poorly contrasted with its visual surroundings.	1.85	1.1	1.2	1.3	1.5	3	3.5	6

Implications for Interaction with Connected Health Devices

During interaction with connected health devices, the loss of visual sensitivity and acuity can lead to difficulties for the older adult when:

1. Discriminating colours and contrast on a screen, particularly in low luminance settings.
2. Reading small, decorative or poorly weighted fonts.
3. Distinguishing between similarly shaped software icons on screens or icons on labels.
4. Coping with glare on a screen or maintaining concentration when glare from external sources are present in the environment.
5. Reading scrolling text.
6. Taking in information from a large field of vision, lack of peripheral vision could have implications for flashing warnings.

#### 3.1.2. Hearing

The decline of auditory function in relation to age is well documented [34,35]. In the U.S.A., 1 in 6 adults report hearing problems while for people aged 75 years or older this rate rises to 1 in 2 adults [36]. Hearing loss has been linked to fall risk [37] and to cognitive decline [38]. Auditory function is generally measured by the subjective behavioural measurement of hearing threshold. Pure-Tone threshold averages are measured over a range of frequencies and reported as the average minimum pure-tone sound heard in the better ear without background noise. This threshold increases with age, indicative of hearing loss and expressed in terms of Decibel Hearing Level (dB HL) at a specific frequency. Kiely *et al.* studied changes in hearing acuity over a period of 11 years and their results are summarised in Table 5 [39].

**Table 5:** Pure-Tone thresholds hearing level (dB HL) at a range of frequencies. Increases in Pure-Tone thresholds hearing level indicate loss of hearing acuity.

At Frequency (kHz)/Age Group (Males/Females)	Young Normal (20 y M)	55–64 years		65–74 years		75–84 years		85+ Years	
		M	F	M	F	M	F	M	F
0.5	7	10	10	12	15	25	28	30	35
1	5	10	10	15	18	27	28	35	35
2	3	15	12	23	20	35	35	45	45
3	4	29	19	39	28	48	40	60	50
4	5	35	21	42	30	58	45	90	58
6	8	45	35	55	45	70	60	85	72
8	10	45	38	63	55	78	70	93	83

Implications for Interaction with Connected Health Devices

During interaction with connected health devices, the loss of audio sensitivity and acuity can lead to difficulties for the older adult when:

1. Perceiving beeps or alarms that reside above 2 kHz.
2. Perceiving low amplitude beeps or alarms.
3. Discriminating acoustic cues that are short in duration.
4. Perceiving verbal feedback that is not clear and reasonably paced.
5. Trying to localise sounds.

#### 3.1.3. Touch Sensation

A tactile threshold is the point at which an external stimulus registers a response in the user and thus is a critical perception in the user experience. As a person ages, the tactile thresholds of various modalities such as light touch, vibrations sense, spatial acuity and pain are increased [40,41]. Of particular importance is the tactile threshold at the fingertip. Deterioration of spatial acuity at the tip of the finger has implications for interaction with connected health devices. It affects the ability to discriminate tactile gaps and bumps as well as the orientation and direction of lines or surfaces [42]. There is a correlation between decrease in tactile threshold and loss of functional dexterity in the hand [43]. This will be addressed in more detail in Section 3.2.1.

Implications for Interaction with Connected Health Devices

During interaction with connected health devices, the loss of sensation and fine motor control can lead to difficulties for the older adult when:

1. Attempting to manipulate small interface components such as buttons, knobs, levers and battery compartments.
2. Perceiving stimuli such as vibration feedback.
3. Distinguishing between tactile gaps, bumps and surfaces.

### 3.2. Psychomotor Performance

Psychomotor performance refers to the performance of cognitive based motor control, particularly finer motor control of the upper limbs such as grip, dexterity, coordination, manipulation and mobility. These psychomotor functions are critically important when using small handheld devices. The decline of psychomotor functionality as a person ages can be measured in terms of loss of muscle power, a decrease in range of motion of joints and an increase in the variability of finer motor movements brought about by motor disorders.

#### 3.2.1. Hand Functionality

The hand is an important functional tool in interacting with a connected health device. It is responsible for pushing buttons, sliding switches, turning knobs, manipulating clips and catches and a host of other functions. The ability to easily manipulate and control a device is an absolute necessity for the device to adhere to a high standard of HCD. The device must create appropriate demands on the hand. This management of demands becomes an even more critical issue when the older adult hand is involved. A reliable and valid objective parameter of the functional integrity of the hand is grip strength [44]. There are two types of functional grip, the power grip and the pinch grip. The power grip is employed with the hand is grasped around an object, like holding the handle of a frying pan. The pinch grip is when the fingers are on one side of the object and the thumb is on the other, like when holding a pen [45]. The change in the strength of these grips as one ages is well documented [46,47] and is summarised in Table 6.

**Table 6:** Mean power grip and pinch grip strength (Kg). D is the dominant hand and ND is the Non-Dominant hand.

Component	30–34 years		55–64 years		65–74 years		75–84 years		85+ years	
Gender	M	F	M	F	M	F	M	F	M	F
Power Grip Strength (D)	55	33.8	50	30	42	27.5	33	22	22.4	16.9
Power Grip Strength (ND)	52.5	32.6	49	29	41	27	32.5	21	23.2	16.7
Pinch Grip (D)	9.9	6.9	10	6.8	8.5	6	7.4	4.8	5.4	3.1
Pinch Grip (ND)	9.3	6.7	9.5	6.5	8.2	5.75	7	4.2	5.5	2.8

A comprehensive analysis of age-induced changes in handgrip and finger-pinch strength, ability to maintain a steady submaximal finger pinch force and pinch posture, speed in relocating small objects with finger grip, and ability to discriminate two identical mechanical stimuli applied to the fingertip was carried out by Ranganathan *et al.*, [48]. They compared the functional performance of the hand between a healthy independent young group and an older adult group (See Table 7).

**Table 7:** Hand functionality. Comparison between a healthy independent young population and older adult group [45].

Component	Definition	Measured by:	Findings
Grip Strength	Main grasping grip	Hand Dynamometer: 3 trials	Older subjects hand grip was 30% weaker (*p* < 0.001)
Maximum Pinch Strength (MPF)	For picking up and holding items	Load cell which measured forces between 0–50 pounds	Older subjects MPF was 26% lower (*p* < 0.05)
Pinch Force Steadiness	Ability to maintain a sub maximal grip for a prolonged period is important for the manipulation of and interaction with everyday objects	Subjects asked to use the load cell to maintain forces at 5%, 10%, 20% of their MPF for a set time	Older subjects were less able to maintain a steady force and their results showed more fluctuations
Precision Pinch Steadiness	Steadiness of the hand while an object is held in the pinch precision	Holding a probe in holes of various sizes the subject was asked to hold the probe without touching the sides of the hole for 20 s. Errors were recorded	Elderly men made 10 times as many errors as younger men (*p* < 0.001) while elderly women made 22 more errors than younger females. This shows a large decline the ability to hold in place a steady pinch
Hand eye Coordination/Hand Dexterity	The ability to coordinate hand movement and the movement of the individual fingers in the necessary configuration to complete tasks	Using one hand, the subject picks the pegs up off the table and places them into the holes on the board, starting with the top left hand hole and completing the board on a column by column basis. This is timed to completion.	Older subject needed 19% more time to complete the peg test (*p* < 0.001)
2 Point Discrimination	The minimal interstimulus distance required to perceive two simultaneously applied skin indentations as two distinct stimuli. Important for tactile feedback during interaction.	A 2 point aesthesiometer is placed on the index finger and the subject is asked whether they can feel one or two points. The variable is the minimum distance between the two points at which the subject can discriminate two distinct points.	Older subjects needed twice the distance to discriminate the two points of the aesthesiometer (*p* < 0.001)

As well as these functional components, the loss of flexibility in the joints of the lower arm, particularly the wrist leaves older adults vulnerable to cumulative and repetitive strains. The range of motion (ROM) of the wrist declines steadily as a person ages. For example, a person aged between 70–79 can expect to have a decreased wrist flexion, extension and ulnar deviation of approximately 10%, 30% and 10% respectively compared to people aged 25–30 (Table 8) [49]. Vulnerability to repeated movement stress is reinforced by the finding that older adults make more hesitant and less fluid movements than younger people. This increases the number of sub-movements during motion adding to the potential risk of repetitive strain [50].

**Table 8:** Range of motion (measured in Degrees) in different age groups. Lower numbers indicate lesser range of motion in the wrist.

Movement	16–30 Years	60–69 Years	70–79 Years	80–89 Years	90+ Years
Flexion	68.6	61.88	61.25	56.50	48.25
Extension	63.6	44.88	44.66	43.55	40.25
Ulnar Deviation	40	39.88	36.08	35.86	29.50

#### 3.2.2. Arthritis and Hand Anthropometry

Arthritis is the greatest contributor when considering limitation of hand functionality. The prevalence of arthritis among older adults is increasing and it limits performance in a wide range of daily activities [51]. Apart from compounding the decline of functionality which we have already discussed, it can make holding or manipulating large objects independent of wrist range of motion in one hand uncomfortable. This is particularly relevant for connected health devices. Anthropometric data might provide useful guidance for the design of containers for users with arthritis. Deformities in the hand caused by rheumatoid arthritis, an extremely common form of arthritis, will affect the interaction a user has with the device. Table 9 shows the maximum grip diameter for individuals with and without dexterity related disabilities such as arthritis [52]. Although the definition of grip diameter used in this study does not completely apply to connected health devices, it is interesting the note the difference in values between a normal healthy subject and one who is suffering from dexterity impairment such as arthritis.

**Table 9:** Comparison of Maximum Grip Diameter (mm) with and without dexterity impairments. Maximum grip diameter is defined as the maximum diameter of a cylinder that a person can grasp with contact between the thumb and middle finger.

	Gender	5th Percentile	50th Percentile	95th Percentile
No Dexterity Impairments	Male	45	52	59
	Female	43	48	53
Dexterity Impairments	Male	34	40	47
	Female	34	40	48

Implications for Interaction with Connected Health Devices

During interaction with connected health devices, the loss of psychomotor strength, dexterity and sensitivity can lead to difficulties for the older adult when:

1. Pressing buttons which require a deal of force that exceeds the capability or comfort of the user.
2. Attempting to press buttons which are close together or are small in surface area.
3. Gripping heavy or cumbersome objects, particularly in one hand.
4. Attempting to reach with the thumb across an interface to manipulate controls when holding a device in one hand.
5. Making certain gestures when interacting with touchscreens (*i.e.*, pinches and swipes).
6. Attempting to attach a device component with one hand without supervision (*i.e.*, cuff on a blood pressure monitor).

### 3.3. Cognitive Performance

While there is a known association between aging and reduction in cognitive performance, there is naturally some debate as to when this change begins [53,54]. Cognitive decline has been shown not just to be a function of age but also a function of past experience, environment, social situation and education level [55,56]. There is little accurate quantification of the true rate and prevalence of cognitive decline [57,58]. In a longitudinal study, Singh-Manoux *et al.* observed certain cognitive processes of five baseline age groups [59]. Subjects were re-tested 10 years later and there cognitive ability rated as percentage change for their original baseline values. Tests included inductive reasoning, short term memory, phonemic fluency, semantic fluency and vocabulary. They found that average performance in all cognitive domains except vocabulary declined across all age groups (See Table 10).

Implications for Interaction with Connected Health Devices

During interaction with connected health devices, the change in cognitive functionality can lead to difficulties for the older adult when:

1. The display and interface is cluttered or overly complex.
2. Feedback is not presented clearly or intuitively.
3. There is no adequate labelling or instructional support.
4. Manipulating controls gives unexpected results.
5. They are asked to remember difficult or complex operational routines.

**Table 10:** Percentage change in cognitive ability at 10 year follow-up. Each group was their own baseline at initial testing point. A negative number reflects a drop or decline in cognitive ability in the age cohort from their baseline value 10 years previous. A positive number reflects an improvement or increase in cognitive ability in the age cohort from their baseline value 10 years previous.

Cognitive Process\Age Group	45–49		50–54		55–59		60–64		65–70	
	M	F	M	F	M	F	M	F	M	F
Reasoning	−3.6	−3.7	−4.1	−4.3	−5.5	−6	−7	−7	−9	−7
Memory	−2.8	−2.4	−3.5	−3.4	−3.6	−2	−4.2	−4.8	−2.8	−3
Phonemic Fluency	4	4.1	−4.8	−3	−4	−4.3	−4.3	−4.6	−4.5	−4.3
Semantic Fluency	3	3.3	−3	−3.2	−4	−2.5	−4.5	v3.5	−4.5	−5
Vocabulary	Neg	Neg	Neg	Neg	Neg	Neg	Neg	Neg	−1	−1

### 3.4. Psychosocial Factors

The general population can be classified into five technology use categories; Innovator, Early Adopter, Early Majority, Late Majority, and Laggards [60]. According to this classification, late majority and laggards adopt new ideas after the average members of society. Older adults tend to exhibit the characteristics of the latter two classes, the late majority and the laggard. These classes may be more conservative, sceptical, cautious, less educated, isolated, risk averse, traditional, and suspicious of innovations. Although it is clear that technology has a potential to play an important role in promoting independence and improving quality of life among older adults, negative perceptions to technology often prevent the adoption of new technology in this population group. Older adults are less likely to use technologies that are perceived to be less beneficial and more difficult to use. When it comes to common technologies such as the internet, it has been found that older adults are more unwilling, unable or afraid to use them than the younger population [61]. The same has also been found for assistive technologies [62].

The connection between emotional factors and technology acceptance for older adults has been studied [63,64]. Most conclusions are born from qualitative based research which is effective if studied and used properly. An excellent example of a qualitative study of the older adult’s emotional response to technology was carried out by Kyung o Kim as part of a doctoral dissertation [65]. The study explored how older adults interact with different technologies and looked to increase understanding of factors influencing their emotional and perceptual responses. Three major themes emerged from the interview based analysis; (1) Simple is Better; (2) Complex Works for Some and (3) Why Do I Need this? Users who follow these themes often share similar characteristics and the study reached some interesting conclusions. Firstly, people with rich networks of support from friends and relatives were more likely to embrace complex technology, while people who were isolated or lacking support preferred simpler technology. The conclusion stressed that the social network of the potential user has a profound effect on their perception of technology. Secondly, compatibility of the technology with one’s goals and lifestyle appeared to have a major influence on acceptance. Just because a technology was perceived to be useful or easy to use, did not necessarily translate to the user wanting to use it, especially if it did not fit in with their personal goals. Finally, the term trialability was brought into the discussion, a term originally introduced by Everett Rodgers in his 2003 book Elements of Diffusion [60]. Trialability can be defined as the perceived degree to which an innovation may be tried on a limited basis, and is related to acceptance. Many older people may not be exposed to or have access to new technologies to try them out which may explain why overall technology acceptance is less in that population group [66]. The study noted that while many people who enter retirement homes or communities may increase their social network among fellow retires, their exposure to technology from more tech savvy family and friends will decrease leading to only small windows of trialability and therefore decreased chance of acceptance.

**Figure 4:**
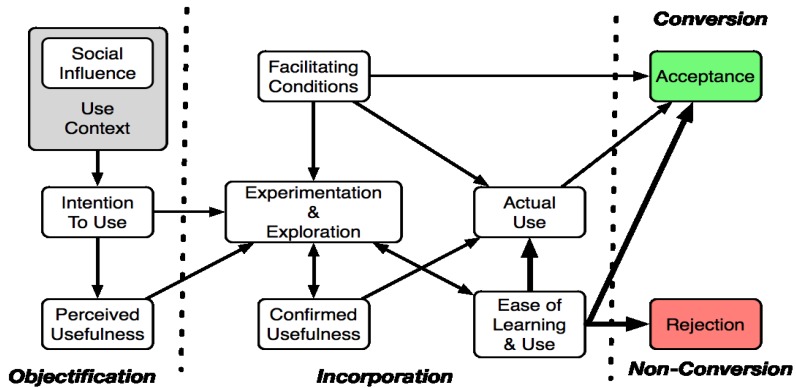
Senior Technology Acceptance Model (STAM).

Adoption of information technology has been shown to vary greatly with the specific experience of the individual [67]. Self-actualisation and realising one’s potential is also an important factor. The confidence with which one approaches a new technology is greatly influenced by cognitive abilities. More recent research has reported that the older subjects took more time to recover from a failure and get more anxious when the tasks are getting more complex [68]. A technology acceptance model specifically designed for older adults, known as the Senior Technology Acceptance Model (STAM), attempts to show the relationship between these factors and technology acceptance (Figure 4) [69]. 

The STAM model consists of three phases; objectification, incorporation, and non-conversion. The objectification phase is influenced by social factors, social and user context and perceived usefulness. The STAM model goes some way to bridging the link between intention to use and actual use by introducing an incorporation phase. The incorporation phase takes experimentation and exploration into account as dynamic factors. Facilitating conditions, confirmed usefulness and perceived “ease of use” are also shown to influence actual use. Facilitating factors, experimentation and exploration show the influence trialability can have on technology acceptance. In the conversion/non-conversion phase, potential users will accept or reject a given technology. The STAM model is meaningful because the model targets older users who may have unique needs, capabilities, preferences, experiences, and limitations as distinct from young adults.

It is now possible to summarise some of the reasons from a psychosocial aspect, why an older adult may not accept the use of connected health devices:

1. Previous Technology Experience: Lack of familiarity or previous experience with similar devices can cause the older adult to dismiss the device or not be aware of its potential use (no perceived usefulness).
2. Complexity: Device is perceived to be too complex (no perceived ease of use).
3. Trialability: Lack of opportunity to use the device experimentally or lack of exposure to new devices in social context.
4. User Context: The use of the device does not fit in with lifestyle or personal goals.

Social, environmental and emotional factors could play a major part in connected health acceptance. Compatibility with personal goals and with current lifestyle may be the most crucial factors. A thorough understanding of older adults’ usage and perceptions of connected health devices, as discussed here, is essential for maximizing the potential that the devices offer, facilitating independence in the users’ everyday life.

## 4. Design Approach and Design Specifications

We have presented the common features of typical connected health devices as well as their typical scenarios of use. We have also summarised the perceptual, cognitive, psychomotor and psychosocial traits of the older adult, a key target group for connected health devices. Given the information presented on the older users capabilities and normal ageing related decline in many of these capabilities, it is possible to make recommendations both in terms of design approach and design specifications for connected health devices.

### 4.1. Design Approach for Connected Health Devices

With such a wide range of technology related capabilities and preferences exhibited by the older adult, it is important that device designers employ an approach which focuses on these characteristics early and often throughout the design process. The best way to achieve this is with early and often user testing. Involving the user throughout the design process is the most effective way of employing design solutions which take into account the capabilities and preferences of the user. Table 11 describes the general stages in the design lifecycle of consumer products, as per Karowski and Stanton 2011 [70]. The process is most fluid at the start, but as it progresses there are fewer opportunities to make design changes. From a HCD perspective, Stage 1 should identify the high priority user needs which the device must meet e.g., Can the user attach a blood pressure monitor on oneself (one handed) and activate the device to detect, record, and display the reading? In Stages 2 and 3 the needs (from Stage 1) are embodied in functionality of the device through its design. Human factors methods are applied at this Stage 1 and Stage 2, to best fit the user’s abilities (perceptual, cognitive, and psychomotor) to the device demand through control and display design (See Figure 4). It is preferable to start performing usability testing in Stage 2 using low fidelity prototypes as changes are relatively cheap to make at this point. By Stage 3 there should be comprehensive usability testing.

**Table 11:** Design lifecycle and methods to apply for design of connected health devices.

Design Stage	Description	Example of approach to use for connected health devices
1 Conceptual Design	The concept for the design is proposed with few decisions made about the embodiment of the device	Ethnographic research to observe users in their own environment performing analogous tasks to that of the planned product Focus groups and interviews with users to elicit intelligence about their needs for a planned device. User diaries where they record notes on a daily basis about their current experiences of a medical condition or the treatments/monitors they use
2 Formalisation	The idea becomes more formal with decisions being made on technical features and functionality. The opportunity for design changes reduce considerably.	Heuristic checklists for good design of interfaces for older users Usability tests (e.g., think aloud protocol) with low fidelity prototypes of the device. Participatory design where users give input on their preferences for the device.
3 Design	The design is finalised and a plan is made for the product development.	Formal usability tests in a lab environment or preferably in the users home
4 Prototyping	Virtual prototypes from CAD models are converted to physical prototypes using 3-D printing or other methods for testing. Only critical changes to the design are often accommodated at this point, especially if tooling has been commissioned.	Usability testing with near identical models of the device. Interfaces might be replicated using off-the-shelf technologies.
5 Commissioning	The final design is produced and released on the market.	Few if any features can be changed at this point. It might be possible to change software through online updates.
6 Operation and Maintenance	The device is in use and supported by the manufacturer (if necessary).	Ethnographic testing of the current device to feed into the next generation of the device.

By Stage 4, prototypes, mock-ups and interface card models should be presented to end users. At this point, the window for making major changes to the design is closing and the designers should have already gathered enough information from the testing in previous stages to produce mock-ups that are extremely close to the end solution. By Stages 5 and 6 the final design solution should have been produced and sent to market and only minor changes can be made in the form of software updates, new accessories, adaptable components or instructional updates, with feedback on device usage feeding into next generation devices.

The design life cycle seen in Table 11 recognises the role and the input of the user early in the design process. This is the basis of Human Centred Design (HCD), a design concept which asks designers to understand the needs and capabilities of the likely users. This implies that the designers can find selected representative users and obtain descriptions of their needs as well as getting them to participate in development teams [29]. The consensus in the HCD community is that there is no way to know in advance which are the particular attributes of a device or service that would make it optimally usable by a target user provided the variety of user profiles and contexts of use. Involving the target users in the product engineering is the optimal approach to assuring that the product will properly meet their needs and fit with their capabilities. HCD represents an alternate methodology to a traditional design approach based on heuristic guidelines and is based on the following four principles:

- Early Focus on Users: Designers should have direct contact with intended or actual users via interviews, surveys and participatory design. The aim is to understand users’ cognitive, physical, attitudinal, and anthropometric characteristics—and the requirements of the jobs they will be doing.
- Integrated Design: All aspects of usability and human factors (e.g., user interface, help system, training plan, and documentation) should evolve in parallel, rather than be defined sequentially, and should be project coordinated.
- Early And Continual User Testing: The optimally feasible approach to successful design is an empirical one, requiring observation and measurement of user behaviour, careful evaluation of feedback, insightful solutions to existing problems, and strong motivation to make design changes.
- Iterative Design: A system under development must be modified based upon the results of behavioural tests of functions, user interface, help system, documentation and training approach. This process of implementation, testing, feedback, evaluation, and change must be repeated iteratively to improve the system.

**Figure 5:**
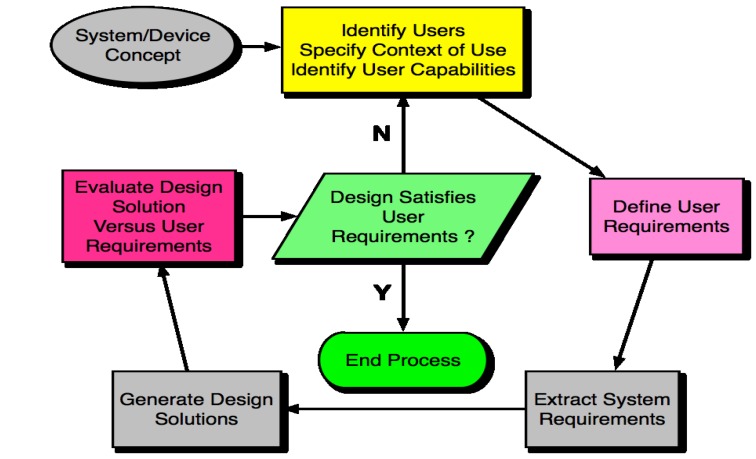
The Human Centred Design Process. The cyclical nature of the process allows for several iterations to take place before a final solution is produced.

The life cycle of HCD, in adherence with the principles outlined above, is shown in Figure 5. This is the design process which should be followed for connected health devices. To achieve a high level of HCD, a final design solution should not be considered to have conformed to HCD until at least three iterations have been carried out, as per the cyclical process seen in Figure 5.

Companies and organisations should be aware of the HCD process and incorporate as a culture within their business. Iteratively, a variety of policy considerations are involved in the adoption of the proposed HCD process. Policies encouraging or incentivizing the adoption of this approach would accelerate the use. Conversely, development of products with this sort of orientation in turn impact the policy related to the deployment of these technologies.

### 4.2. Design Specifications

Apart from the HCD concepts outlined above, there are also specific steps designers can take to ensure that connected health devices conform to a high level of usability and human factors for the older adult. There are a number of general guidelines which should be followed before specific design features are considered.

#### 4.2.1. Display

The display is one of the most important output features on a connected health device. In Figure 2, we saw how the display is the interface at which device output is perceived so that it can be acted upon. As such, the design and function of displays will directly contribute to the user experience of the device. We have outlined a range of screen types typically encountered in popular connected health devices (Table 12).

**Table 12:** Comparison of character sizes and display types for popular connected health devices.

Device	Display Type	Main Characters (h × w)	Approx. Font Size (pt)	Secondary Characters (h × w)	Approx. Font Size (pt)	Margin/Header Characters (h × w)	Approx. Font Size (pt)
Omron MIT Elite (Blood Pressure Monitor)	LCD Black and White	20 × 12	56	12 × 8	34	1 × 2	3
Omron HJ-720ITC Pedometer	LCD	4 × 2	11.3	-	-	-	-
Spirodoc Spirometer	LCD Backlit Touch screen	4 × 4	22	-	-	-	-
Prodigy Autocode Talking Metre	LCD	22 × 7	62	-	-	2 × 1	6
Gentle-Temp from Omron	LCD	8 × 3	22	-	-	-	-
ChoiceMMed Pulse Oximeter MD300C21	Dual colour OLED	7 × 4	20	-	Na	2 × 2	6
HCG-801-E from Omron (ECG Metre)	Graphic LCD high resolution screen with backlight	4 × 2	11.3	-	-	2.5 × 1	7

Connected health devices are used primarily indoors in the home but may also be used outdoors. For passive LCD screens, common to many devices, lighting levels in the home may often not be adequate for reading comfortably from the screen while in outdoors environments there are many sources of glare. We know from the information in Table 4 that both normal low contrast acuity (LCA) and low contrast acuity in glare (LCAG) will have diminished by a factor of at least 1.5 from the baseline for a typical 70 year old. However from studying the same data we find that high contrast acuity (HCA) will only have diminished by a factor of 1.1. Therefore it is important to incorporate a screen type that not only has low glare and a backlight option but that allows for high contrast between characters and background. The screen should also afford a wide viewing angle. The information on the screen may be read while the user is lying or sitting down with hands by the side. The user should be able to comfortably view and comprehend screen information from a variety of angles. This means that older models of LCD screens should be avoided. Warning information propagated from the screen should repetitively flash rather than having it appear statically although it should not flash so fast that reading is impaired. Again, backlights can play an important role here as their flashing can be an easy way to grab the attention of the user.

#### 4.2.2. Character Size

Even with the increasing use of icons on screen interfaces, much of the critical information of connected health devices is presented in text and numerical format. It is clear that text and numerical characters are dominant informational features on connected health devices. When it comes to reading characters on a display, there are two important aspects for HCD; legibility and readability. Legibility is more relevant in terms of human factors, in that is determines how easy individual characters are to read. This depends on size, weight and colour among other factors. Readability is defined as how easy it is to read a body of characters, which can depend on layout, justification and colour tone. While optimum character font sizes for the older adult user have not been agreed upon in literature, it is clear that there is some definite size limit below which readability and legibility will become impaired (Table 12).

Darroch *et al.* carried out an experiment where speed and reading accuracy was measured for fonts between 2 and 16 point for both older and younger users [71]. They found that above 6 point font there was little difference in objective performance but subjectively older users preferred a slightly larger font with the optimum and most comfortable range being an 8–12 point font size. Kroehmer *et al.* have also given recommendations for character size when the user is at various distances from the display (Table 13) [72].

**Table 13:** Recommendations for display characters from the handbook of occupational ergonomics.

Distance of Display from Eye (mm)	Height of Lettering	Approx. Font Size	Width of Lettering
Up to 500	2.5	7	1.875
501–900 (Typical arm length)	5	12–14	3.75
900–1800	9	20–25	6.75

The recommendations in Table 13 are particularly relevant to connected health devices, given that they are handheld and the user would typically hold them at a comfortable arm’s length from the face. The readability of text also depends on contrast and luminance. Table 14 shows the relative letter sizes required under different levels of contrast and lighting conditions for two different older age groups [21].

**Table 14:** Recommended minimum optimum text size and weight under different conditions as provided by a SKI study on older adult vision [21]. There is a sharp difference between optimum character size for a user aged 62 and a user aged 87. Ages are averaged for the two groups studied.

Age	Bright light high contrast	Font Size	Bright light low contrast	Font Size	Dim light low contrast	Font Size	Glaring light low contrast	Font Size
62.5	m	7.5	m	12	m	13.5	m	18
87.5	m	12	m	18	m	24	m	36

Many of the connected health devices currently on the market have high contrast LCD screens although many of them are not backlit which may mean readability of characters is dependent on background lighting.

#### 4.2.3. Touchscreens as Displays

The recent evolution of touchscreen means that button size, button layout and font size are customisable. The touchscreen also represents a more intuitive interface as the user is directly interacting with the device controls. However the touchscreen presents its own challenges. For the older adult the touchscreen must have a greater tolerance for error than with a normal user and must not rely on fast or rapid hand movements to carry out functions. The traditional user actions needed to interact with a touchscreen include taps, pinches, swipes and drags. These actions may be problematic for older adult users who suffer from chronic pain or lack of flexibility in the joints of the hand as discussed in Section 3.2. In a study of how the older adult interacts with a touchscreen interface it was found that while older adults are slower than the younger age group they are not much less accurate even when it comes to more complex gestures [73]. Their results showed. They were effectively able to retrace complex patterns accurately, regardless of the three screen sizes presented. Although speed of gesture was slower in the older cohort than the younger cohort, this was not noted as a critical downfall as in some cases it actually prevented errors that the younger cohort were susceptible to.

A similar experiment carried out by Kobayashi *et al.* found that while older adult users improved dragging and pinching performance time by as much as 25% from one week to the next in a two week experiment, tapping small objects was a major problem [74]. Users often tapped outside the target area and introduced error reduction strategies such as exerting more pressure on the screen, carrying out multiple taps to ensure the target was hit or holding their finger on the screen longer than necessary, often confusing the system into initialising a drag or hold command. Sometimes the finger blocked the small target so that the user could not tell if the colour of the target had changed to signify a successful press.

The space between two or more touch sensitive areas is as a factor that could influence user experience. Spacing is a trade-off between button size, desired accuracy, desired reaction time and display size. Jin *et al.* found that using excessive spacing decreased reaction time as users had to spend more time searching the screen [75]. They found the optimum spacing between adjacent elements to be 6.35 mm for older adults. Their findings closely correspond to ISO recommendations, which states that a minimum spacing of 5 mm should be used [76]. Colle *et al.* reported that 1 mm space could be used if the screen has severely limited space [77]. In cases of very limited screen area, 0 mm space can be used without effect responding time although it decreases accuracy and lowers user satisfaction. For capacitive touchscreens, used in most modern smartphones, the need for excessive spacing between elements decreases due to the quality and sharpness of the screen. Big buttons also negate the need for excessive spacing.

#### 4.2.4. Buttons/Switches

Buttons are an almost unavoidable feature of connected health devices. Even if a touchscreen is incorporated into the device, buttons may still exist to control volume, locking, on/off, syncing and alarms. Even on most modern smartphones there generally exists a physical on/off button as well as a multipurpose “home” button. Buttons can be considered a weak part of any device. Due to the constant mechanical stress they are often the first part of the interface to breakdown. Poor button design can directly contribute to a negative user experience as we have discussed in Section 1 (Common personal Connected Health Devices, Table 2). There are several design specifications than can allow buttons to become a seamless part of the interface. It goes without saying that any kind of button that requires twisting or an uncomfortable level of manipulation should be avoided. Table 15 provides a summary of the button size measurements.

**Table 15:** Comparison of the button surface area between the connected health devices analysed.

Device	Main Button	Button Area	Secondary Buttons	Button Area
	(h × w mm)	(mm^2^)	(h × w mm)	(mm^2^)
Omron MIT Elite (Blood Pressure Monitor)	16.5 × 41 (power)	676.5	25 × 11 (function)	275
HJ-112 Pedometer	10 × 8 (Mode)	80	8 × 6 (Memo); 4 × 4 (set)	48; 16
Spirodoc Spirometer	27 × 7 (Power)	189	na	na
Prodigy Autocode Talking Metre	10 Diameter (Power)	78.5	na	na
Gentle-Temp from Omron	15 × 20 (Power)	300	na	na
ChoiceMMed Pulse Oximeter MD300C21	5 mm Diameter (Power)	19.25	na	na
HCG-801-E from Omron (ECG Metre)	10 Diameter (Power)	78.5	6 × 10 (Side Function)	60

Jin *et al.* found that reaction time decreased with increased button size although it was unclear whether accuracy significantly increased with button size [75]. They found, consistent with other studies, that optimum button sizes resided between 250 mm^2^ and 360 mm^2^. Recommended button sizes, button travel, required press force and distance between buttons were also discussed by Kroehmer *et al.* (Table 16) [72]. Buttons are an important feature of many interfaces, connected health devices being no exception. Accordingly, the design of connected health devices for the older adult should consider issues such as dexterity and repetitive strain.

**Table 16:** Recommended push button characteristics.

Button Characteristic	Least Required Value
Surface Area	110–175 mm^2^
Surface Area (for an emergency button)	700–1250 mm
Travel (distance button must be pressed to trigger function)	3–10 mm
Spacing Between Buttons	20 mm
Force Required for Operation	2.5–5 N

#### 4.2.5. Audio Feedback

Audio output is primarily used to convey feedback information to the user. The obvious first consideration when designing audio systems on a connected health device is to design for adjustability. This specifically refers to volume although it is also a concern that the user may accidentally turn the volume down too low or off altogether, thereby negating the usefulness of reminders, notifications and alarms. Audio feedback can be combined with tactile feedback like vibration and a flashing screen to ensure that feedback is not exclusively dependent on volume and frequency of audio signals.

From our analysis of the older adult user’s auditory response thresholds, it is clear that frequencies above 3–4 kHz cannot be as easily picked up by the older adult ear. Therefore it is recommended that important auditory feedback reside in the range of 500–1000 Hz with an adjustable volume setting. If voice feedback is used, similar sounding terms should be avoided.

#### 4.2.6. Module Size

An important consideration for hand held devices is the size of the device itself in relation to the hand which will be holding it. This becomes especially important for devices that have buttons and switches that may need to be manipulated with the holding hand while the other hand is engaged in another task. This characteristic of a hand held interface is known as reachability. The issue of reachability has come into focus recently with the release of the iPhone 5, which has a screen size of 4 inches compared to the 3.5 inches of the iPhone 4. Apple has said that this increase is possible due to a 20% reduction in the phone thickness, thereby still affording the same grip diameter as the previous model. 

It is interesting to compare these data with information in Table 8 and Table 9 and how the dimensions and weights might affect the reachability of users suffering from conditions which affect the anthropometry of the hand. The care which Apple takes in assuring that the iPhone is completely useable in one hand is an example which should be followed in the design of connected health devices. Table 17 shows the varying dimensions and weights of common connected health devices.

**Table 17:** Size and weight or common connected health devices.

Device	Module Size (hXwXd)	Weight (g)
Omron MIT Elite (Blood Pressure Monitor)	157 × 74 × 34	270
Blood Glucose Monitor	96 × 52 × 22	55
Pulse Oximeter	58 × 32 × 34	28
Pedometer	73 × 47 × 17	35
Spirodoc Spirometer	73 × 53 × 16	116
Weighing Scales	101 × 48 × 16	2870
GentleTemp	94 × 45 × 58	50
HCG-801-E from Omron (ECG)	121 × 67 × 24	130

### 4.3. Design Recommendations Summary

In Section 4.2 we have detailed the key common feature sets (display, character size, buttons/ switches, audio feedback and module size) of many connected health devices in the context of the capabilities of the older user. It is now possible to make some standard recommendations for the design of connected health devices for the older adult. The specifications presented in Table 18 should be where the design specification standard for connected health devices for the older adult begins. Naturally during the design process, user testing will give the designer the opportunity to customize and optimize these specifications based on the feedback received.

**Table 18:** Design Recommendations for common feature component of connected health devices.

Feature	Recommendation	References
Screen Type and Screen Lighting	Low quality LCD screens will often display dull tones and have extremely narrow viewing angles, making it hard for a user to see details on the screen if they are looking at them off centre. This can be avoided by either increasing the screen size or installing higher quality LCD and OLED screens in devices, allowing sharper detail and wider viewing angles.	[21]
Colour	The effects of ageing on colour vision perception may significantly diminish the visual effectiveness of certain colour combinations. Make critical elements larger and ensure that they have high luminance contrast with their surroundings. Warnings should not be solely dependent on colour, but also on visual cues such as flashing, labelling and positioning.	[21]
Character Size	With the advent of touchscreens, adjustable text size will become the norm. Our recommendation is that character size should not go below 12 pt on a High Contrast screen interface.	[21,71,72]
Button Surface Area	Designers should aim for button sizes which allow for easy visibility and easy manipulation. Button surface area should typically reside above 150 mm^2^	[72,75,78]
Required Button Press Force	- Required push force should not exceed 5 N, and should reside between 2.5 N–5 N. - This is consistent with the AMMI Medical Device Standard, which states that the required press force should not exceed 5 N.	[5,72,75]
Touchscreen	Touchscreen are a more intuitive way of interacting with a display, but poor quality touchscreens are no substitute for good buttons and as such designers should be wary of introducing a touchscreen just for novelty sake. The touchscreen has to be of good quality in order to prevent user frustration and has to have a big enough screen size so as to allow for adequate spacing between elements. It has been shown that older adults can interact effectively with touchscreen interfaces.	[73,74]
Spacing Between Buttons and Touchscreen icons	- For touch sensitive elements of touchscreens, the recommendation is to have pacing between elements of no less than 5 mm with an optimum of 6.35 for older adult users to maintain a frustration free level of accuracy. - Smaller spacing, as little as 1mm, is manageable but will add to user frustration and decrease gadget tolerance. - Spacing between buttons should reside around the same distance as for touch sensitive elements, although by using bigger buttons the need for big spacing decreases.	[74,75,76,78]
Audio Output and Feedback	It is recommended that important auditory feedback reside in the range of 500–1000 Hz with adjustable volume level. When designing tones, beeps and alarms the ATH at each frequency must be taken into account for each age group. It is not just a case of making sounds louder, but also taking into account the frequency at which sounds are transmitted. If including voice feedback on a device via a speaker, the clarity of tone must be optimum otherwise users could easily misinterpret similar sounding words. Similar sounding words should be avoided when possible.	[39,79]
Tactile Feedback	- Tactile bumps and distinguishable surface transitions should be used around important interface elements such as buttons and ports. - Vibration should be used for signalling, especially for seeking attention of the user and as a feedback to show that a button has been pressed correctly.	[40,41,42]
Device Size	- Device dimensions must be such that a user can comfortably hold the device in one hand and still manipulate the controls with the thumb on the same hand.	[52]
Reducing Cognitive Load	- The interface should be based on user-oriented, terms and concepts rather than computer concepts. - The system should display an appropriate level of consistency. Commands and menus should have the same format. - If a command operates in a known way, the user should be able to predict the operation of comparable commands. - The system should provide some resilience to user errors and allow the user to recover from errors. - Some user guidance such as help systems, on-line manuals, *etc.* should be supplied	[68]

## 5. Conclusions

In this paper, we have discussed and presented a review of the terminology associated with usability, human factors and user experience and how they relate to interaction with everyday devices. We then reviewed some current market connected health devices, how they are used and what kind of interface features they have in common. We have also specified the user characteristics of the biggest user group of connected health devices, the older adult. We have characterised the older adult user in terms of perceptual, psychomotor and cognitive ability. We have established the common features of connected health devices that may present problems for the older adult user given the older adult populations limited abilities and provided our own design recommendations in terms of design approach and design specifications. In carrying out this analysis, we have effectively met, from a theoretical standpoint, the first two guidelines of Human Centred Design as per the guidelines in ISO 9241-210. The guidelines are as follows:

(a) Understand and specify the context of use: We have specified a user group and analysed the context in which devices are used and how they are used. Having specified the user group, we have analysed their requirements based on quantitative data on their perceptual, psychomotor and cognitive capabilities.
(b) Specify the user requirements: From this data, we produced a set of specific requirements which the design must meet in order to create a degree of fit between device and user; an example of this is seen in Table 18 of Section 4.3.
(c) Produce design solutions: The next step is to produce design prototypes based on these specifications and present them to the user in the form of user testing.
(d) Evaluate: Once feedback has been received, the process begins again until all user requirements have been met.

**Figure 6:**
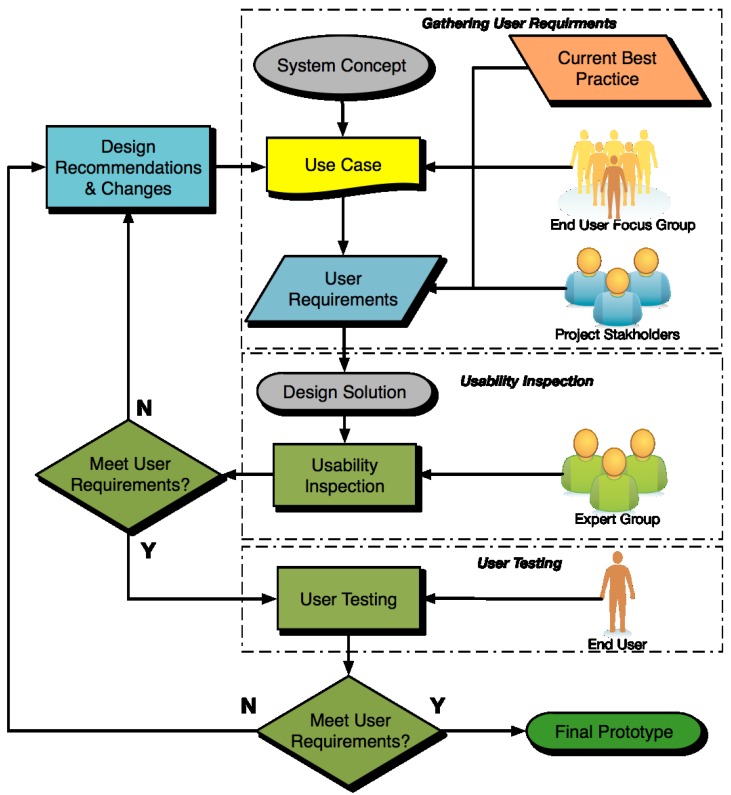
Design Methodology for enhanced Usability.

This paper argues that optimal design for the older adult user group can be achieved by following the HCD process and we propose a design methodology for enhanced usability (Figure 6). The benefits of adhering to these guidelines are that it provides a comprehensive structure for the role of usability, human factors and user experience as part of product quality. The broader concept of quality in use as an ISO standard increases the business relevance of Human Centred Design. Companies and organisations should be aware of the HCD process and incorporate it as a culture within their business [6]. Iteratively, a variety of policy considerations are involved in the adoption of the HCD process. Policy encouraging or incentivizing the adoption of this design approach would accelerate the use of it, while creating a product that follows this approach will have a profound and positive effect on future development. From our review of the connected health devices in this paper, it is reasonable to assume that some older adults may struggle with some aspect of their use. Issues with character size, button size, button layout, interface presentation or audio feedback may be just some of the issues they could encounter. From our review of psychosocial characteristics of the older adult, it is also apparent that this user group may be less likely to have adequate support structures in place to help them operate and maintain these devices. The goal for designers should be to produce devices that need as minimal introduction, maintenance and instructional support as possible. Device design that places minimal demands on as many users as possible, follow a concept known as universal or inclusive design. These devices are designed in such a way that they are flexible enough to be usable by people with no limitations as well as by people with functional limitations related to disabilities or old age [79]. The design for connected health devices should follow 7 principles based on the universal design model [80].

Apart from these design concepts and approaches, the starting point for design solutions should rest on the basics of human factors and usability, which we have established are the main components of user experience. Character size, audio volume, colour tones and button size may seem old fashioned and clichéd, but these are the simple interface characteristics which can greatly influence user experience. We have presented some simple guidelines and specifications which designers should regard as a first approximation to the preferences of the majority of older adult users.

The task for organisations which design connected health devices is clear. They should strive for the implementation of a Human Centred Design approach with explicit involvement of users through the design process. This approach should be embedded in the corporate make-up of medical device organisations. With the number of older adult users increasing, the HCD approach which designs for the vast range of capabilities exhibited by this user group is the most effective and viable way of creating highly acceptable devices.

1. **Equitable Use:** The device should provide the same means of use for all users, identical whenever possible; equivalent when not.
2. **Flexibility in Use:** The device accommodates a wide range of individual preferences and capabilities like those identified in Section 3.
3. **Intuitive Use:** Device interface is easy to understand, regardless of the user’s previous experience with similar devices.
4. **Perceptible Information:** The device communicates necessary information effectively to the user, regardless of ambient conditions or the user's perceptual abilities.
5. **Tolerance for Error:** The device minimizes hazards and the adverse consequences of accidental or unintended actions.
6. **Low Physical Effort:** The device can be used without causing discomfort, fatigue or strain.
7. **Size and Space for Approach and Use:** Appropriate size and space is provided for approach, reach, manipulation, and use regardless of user’s body size, posture, or mobility.
